# Genome-Wide Association Study Reveals Candidate Genes for Litter Size Traits in Pelibuey Sheep

**DOI:** 10.3390/ani10030434

**Published:** 2020-03-04

**Authors:** Wilber Hernández-Montiel, Mario Alberto Martínez-Núñez, Julio Porfirio Ramón-Ugalde, Sergio Iván Román-Ponce, Rene Calderón-Chagoya, Roberto Zamora-Bustillos

**Affiliations:** 1 TecNM/Instituto Tecnológico de Conkal, Av. Tecnológico S/N, Conkal, Yucatán 97345, Mexico; wilber.hernandez@itconkal.edu.mx (W.H.-M.); julio.ramon@itconkal.edu.mx (J.P.R.-U.); 2 Departamento de Ciencias Agropecuarias, Universidad del Papaloapan, Loma Bonita Oaxaca 68400, Mexico; 3 UMDI-Sisal, Facultad de Ciencias, Universidad Nacional Autónoma de México, Sierra Papacal-Chuburna Km 5, Mérida, Yucatán 97302, Mexico; mamn@ciencias.unam.mx; 4 Centro Nacional de Investigación Disciplinaria en Fisiología y Mejoramiento Animal, INIFAP, Ajuchitlán Colón, Querétaro 76280, Mexico; chagoya_91@hotmail.com

**Keywords:** prolificacy, Pelibuey sheep, genome-wide association study

## Abstract

**Simple Summary:**

Reproductive traits are economically important in the livestock industry, and this is of greater relevance when it comes to indigenous animals, since their study allows improving their use and management. Through a genome-wide association study (GWAS), the reproductive trait of the litter size (prolificity) was analyzed in the indigenous Pelibuey sheep. Several single-nucleotide polymorphisms (SNPs) and candidate genes potentially associated with litter size trait were found in the multiparous ewe’s group. These findings help to understand the genetic basis of reproductive traits of hairy Pelibuey sheep.

**Abstract:**

The Pelibuey sheep has adaptability to climatic variations, resistance to parasites, and good maternal ability, whereas some ewes present multiple births, which increases the litter size in farm sheep. The litter size in some wool sheep breeds is associated with the presence of mutations, mainly in the family of the transforming growth factor β (TGF-β) genes. To explore genetic mechanisms underlying the variation in litter size, we conducted a genome-wide association study in two groups of Pelibuey sheep (multiparous sheep with two lambs per birth vs. uniparous sheep with a single lamb at birth) using the OvineSNP50 BeadChip. We identified a total of 57 putative SNPs markers (*p <* 3.0 × 10^−3^, Bonferroni correction). The candidate genes that may be associated with litter size in Pelibuey sheep are *CLSTN2*, *MTMR2*, *DLG1*, *CGA*, *ABCG5, TRPM6*, and *HTR1E*. Genomic regions were also identified that contain three quantitative trait loci (QTLs) for aseasonal reproduction (ASREP), milk yield (MY), and body weight (BW). These results allowed us to identify SNPs associated with genes that could be involved in the reproductive process related to prolificacy.

## 1. Introduction

The sheep meat demand in Mexico is not covered by national production, partly due to the country’s low productive efficiency [1], creating opportunities to intensify lamb production in each region of the country [2]. Hair sheep that guarantee a relatively constant production of lamb throughout the year and the availability of meat [3], such as Pelibuey ewes, predominate throughout the Mexican territory under different types of climates. They are characterized by their rusticity, high adaptability to different environments, excellent maternal ability, prolificacy, and active reproduction most of the year [4,5]. The prolificacy in the Pelibuey sheep is ~1.5 lamb per calving, which could be of great interest to breeders. In different breeds of sheep, it was found that prolificacy is associated with mutations in different genes, which were identified mainly in wool breeds [6,7,8], but few studies were done on hair breeds such as Pelibuey sheep. In this way, several studies showed that genes related to the transforming factor group β (TGF-β), bone morphogenetic protein 15 (*BMP15*), growth differentiation factor 9 (*GDF9*), and bone morphogenetic protein of the receptor-IB (*BMPR-IB)* are essential for normal follicular development in the primary follicle stage in sheep [9,10,11]. TGF-β plays a role in the regulation of oocyte maturation and follicular development, and it is probably involved in cumulus expansion in mice [12], and granulosa cell proliferation (GC) [13]. The single-nucleotide polymorphisms (SNPs) identified in these genes were shown to be associated with an increase of ovulation rate (OR) and litter size [14]. Nine SNPs were evidenced in the gene *BMP15* such as *FecX^I^, FecX^H^* [6], *FecX^B^, FecX^G^* [10], *FecX^L^* [14], *FecX2^W^* [15], *FecX^O^, FecX^Gr^* [16], *FecX^Ba^*^r^ [17], and deletion of 17 base pairs in *FecX^R^* [9]. In the *GDF9* gene, eleven point mutations were reported: *G1, G2, G3, G4, G5, G6, G7, G8* [10], *FecT^T^* [18], *FecG^E^* [19], and *FecG^WNS^* [20]. These mutations are strongly associated with litter size in sheep and are potential molecular markers used in breeding programs to increase productivity and efficiency of lamb meat production [21]. The use of genetic markers can reduce generational time in the genetic selection process through introgression of wild alleles in the population and ensuring a high production on farms. Genome-wide association studies (GWAS) are used for scanning markers across the complete genome to identify genetic variations associated with a particular trait [22]. GWAS are widely used for the detection of single-nucleotide polymorphisms (SNPs) associated with economically important traits, revolutionizing the way to locate regions of quantitative trait loci (QTL). In sheep, they were used to identify markers associated with resistance to parasites [23], selection in locally adapted livestock [24], fat deposition in the tails [25], body weight [26], and prolificacy [9,25,27]. In Ile de France sheep, using Illumina Ovine 50K, four SNPs (s17197, s48166, s25202, and OAR5_47774570) associated with prolificacy were reported in the *GDF9* gene [27]. In four breeds, statistically significant SNPs were reported (rs416717560 and rs421635584 in Wadi, rs429755189 in Hu, rs412280524 and rs401960737 in Finnsheep, and rs423810437 in Romanov sheep) for litter size [25]. However, these studies were conducted on wool sheep, while there is no research on hair breeds such as the Pelibuey sheep. Therefore, the objective of this study is to identify SNPs associated with litter size in multiparous Pelibuey sheep via micro-array analysis using the OvineSNP50 BeadChip, as well as to integrate the analysis of metabolic pathways associated with SNPs. 

## 2. Materials and Methods 

### 2.1. Animals Used and Obtaining Samples

For our study, we used 48 Pelibuey ewes, with record of three consecutive births. Those with two or more lambs per birth were considered prolific sheep (case group, *n* = 24) and those with a single lamb at birth were considered non-prolific (control group, *n* = 24). DNA was extracted from blood samples: eight samples of prolific and six samples of non-prolific sheep were obtained from the sheep farm “El Rodeo” located in Tabasco, Mexico (17°50′39″ north (N) and 92°49′01″ west (W)); seven samples of prolific sheep and 13 samples of non-prolific sheep were obtained from the farm “Las Potrancas” in Tabasco (18°03′04″ N and 92°49′23″ W); five prolific sheep and five non-prolific sheep were obtained from “San Alberto” located in Yucatán, Mexico (20°49′42.09″ N and 89°48′42.29″ W); four prolific sheep were obtained from the farm “El Cortijo” located in Campeche, Mexico (19°43′48.92″ N and 90°05′11.58″ W). Subsequently, the blood samples were stored at −4 °C until DNA extraction.

### 2.2. Extraction of DNA and Genotyping

DNA was extracted from 200 μL of blood, using the Quick-DNA™ Miniprep kit (Zymo Research, Irvine, CA, USA), according to the manufacturer’s protocol. The DNA obtained was purified using the DNA Clean & Concentrator™ -5 kit (Zymo Research). The DNA concentration was measured with a Nanodrop Lite (Thermo Scientific®, Wilmington, DE, USA), and integrity was visualized on a 1.5% agarose gel. The genotyping of the two experimental groups was performed with a medium-density array Illumina OvineSNP50 Beadchip (54, 241 SNPs) in the Illumina HiScan System according to the manufacturer’s instructions, at GeneSeek (Lincoln, NE, USA).

### 2.3. Genotyping Analysis and Data Quality Control

A total of 54,241 SNPs were obtained from the genotyping data. The PLINK v1.07 software [28] was used for quality control (QC). The SNPs that met the following criteria were selected: SNPs that were positioned on chromosome (1 to 27), call rate < 0.95 (--geno: 0.05 and --mind: 0.05), and minor allele frequency (MAF) < 0.02 [28]. SNPs that failed the Hardy–Weinberg equilibrium (HWE) (*p*-value < 0.001) were excluded [29]. Relatedness of the genetic distances between populations was calculated based on a pairwise state identity data (IBS) distance matrix of all samples, for which the first three multidimensional scale (MDS) dimensions were extracted (–genome, –cluster, –mds-plot 4) and visualized with R v. 3.5.2 [30], and all pairs of individuals with IBS > 0.4 were excluded from further analysis. 

### 2.4. Genome-Wide Association Analysis

The GWAS was conducted using PLINK v. 1.09, consisting of a comparison of allele frequencies between cases and controls, with asymptotic and empirical *p*-values available. To control the family-wise error rate (FWER), a Bonferroni correction was used as described by Brinster et al. [31]. The suggestive association significance threshold was *p* < 0.05 ((*p* < 2.67 × 10^−5^) = (*p* < 0.05/(50,602/27)) at the chromosome-wide level [31,32,33]. The significant association indicates that the chromosome-wide level association considered corresponds to a *p*-value less than 10^−3^ [34]. The quantile–quantile (Q–Q) plots were visualized by plotting the distribution of obtained vs. expected log10 (*p*-value) with inflation factors (λ). The association map and the significant SNPs were visualized in the Manhattan plot with a threshold line. The Manhattan and quantile–quantile graphics were plotted with R v. 3.5.2 [30]. 

### 2.5. Candidate Gene and QTLs Associated

The genes identified as associated with significant SNP loci were aligned to confirm their chromosome and physical location, using ovine reference genome OAR_v4.0 with the Genome Data Viewer, available online from the NCBI’s Genome (https://www.ncbi.nlm.nih.gov/genome/gdv/?org=ovis-aries). An SNP was considered to be from a particular gene if it mapped within it. The QTLs were located in the database Jbrowse [35], using online page (https://www.animalgenome.org/) which contains QTLs previously reported in sheep [36,37,38,39].

### 2.6. Analysis of Gene Ontology and Metabolic Pathways

For the gene ontology (GO) enrichment analysis, the genes identified in GWAS were analyzed using the Database for Annotation, Visualization, and Integrated Discovery (DAVID) platform [40], with the sheep genome OAR_v4. In addition, we performed a metabolic pathway analysis using the Kyoto Encyclopedia of Gene and Genomes (https://www.genome.jp/kegg/genes.html) [41]. To graph GO annotations, the WEGO v2.0 program was used [42].

## 3. Results

### 3.1. Genotyping Analysis and Data Quality Control

**Figure 1:**
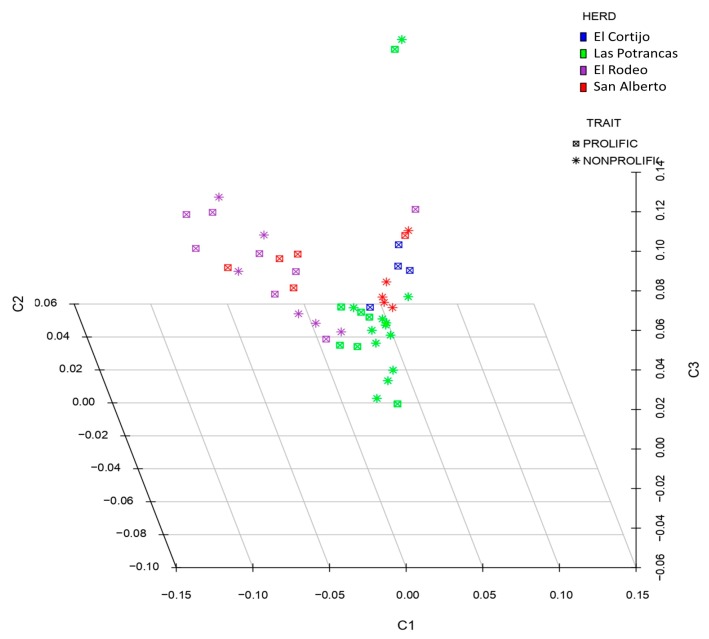
The multidimensional scale (MDS) analysis of genotypes included in this study. The analysis was performed for the first three components (C1, C2, and C3). The color indications for the herd are as follows: El Cortijo, blue; Las Potrancas, green; El Rodeo, purple; San Alberto, red.

After QC analysis, 50,661 SNPs were genotyped. Subsequently, 59 SNPs were excluded from our dataset as they did not pass the HWE and MAF tests. One non-prolific ewe of 24 ewes was discarded for presenting a low call rate (<95%). Finally, 50,602 autosomal SNPs from 47 Pelibuey sheep were used to carry out genotype association tests. The stratification of alleles in the population based on state identity data (IBS) was plotted on the multidimensional scale (MDS) among the same groups of related sheep. The treatments did not show a clear formation of clusters within the populations, as shown in Figure 1. The distribution of the alleles indicates that there is no genetic difference between the two groups, since they belong to the same breed.

### 3.2. Genome-Wide Association Analysis

**Figure 2:**
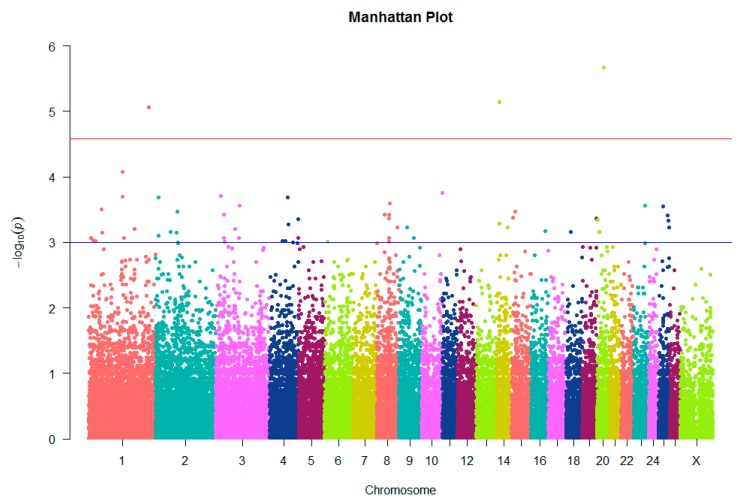
Manhattan plot showing single-nucleotide polymorphisms (SNPs) associated with litter size on the ovine chromosome. The red line corresponds to the 5% chromosome-wide significance threshold using a Bonferroni correction (2.6 × 10^−5^). The blue line corresponds to a suggestive chromosome-wide threshold of 10^−3^.

The association analysis was performed on 50,602 SNPs distributed in 27 chromosomes. Of these, only three SNPs which were defined as associated with litter size passed the Bonferroni test correction at the chromosome genome-wide level and 54 SNPs were only suggestive, as shown in Figure 2 and Table S1. 

**Figure 3:**
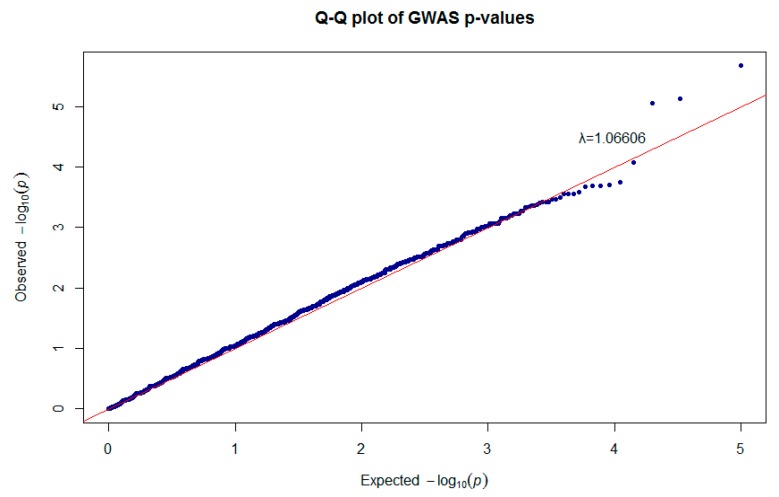
Quantile–quantile plot of genome-wide association study (GWAS) shown in the Manhattan plot.

The quantile–quantile plot shows the total distribution of the observed *p*-values (−log10 *p*-values) of 50,602 SNPs versus the expected values, showing that some deviated from of the expected with an inflation factor (λ) of 1.06606, as shown in Figure 3.

### 3.3. Gene Ontology and KEGG Analysis

**Figure 4:**
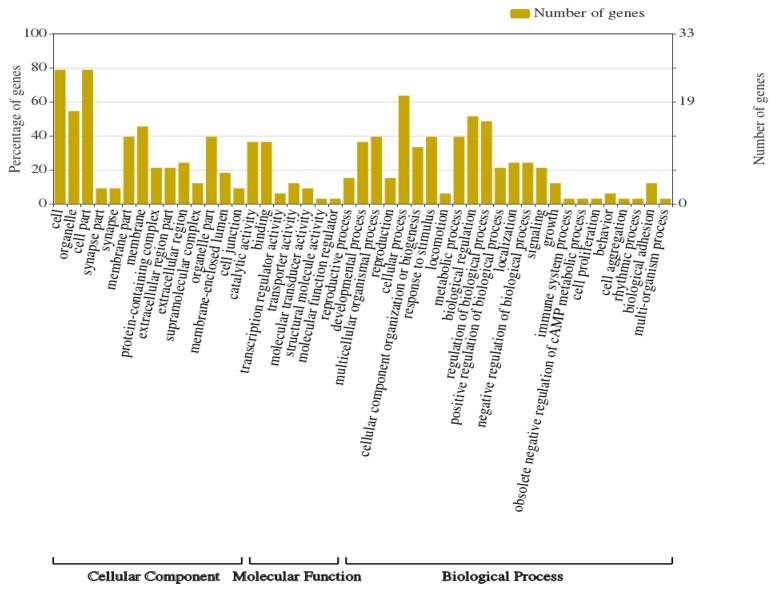
Gene ontology categories identified in genes with significant SNPs.

In order to determine the molecular and metabolic functions in which the 57 selected genes participate, the identification of their ontological categories and associated metabolic pathways was carried out. Identified genes were categorized into 46 functional groups of the Gene Ontology (GO) classification, distributed as follows: 25 functional groups for biological process (BP); 14 groups for cellular component (CC); 7 groups for molecular function (MF). The highest abundance of genes was represented in the CC category (Figure 4).

Among the ontological categories identified in the 57 genes, those related to reproduction were as follows: GO:0001701: in uterus embryonic development (ANKRD11); GO:0008585: female gonad development (ARID5B); GO:0070373: negative regulation of ERK1 and ERK2 cascade (DLG1); GO:0032870: cellular response to hormone stimulus (ROBO2); GO:0061364: apoptotic process involved in luteolysis (ROBO2); GO:0032275: luteinizing hormone secretion (CGA); GO:0032870: cellular response to hormone stimulus (CGA); GO:0046621: negative regulation of organ growth (CGA); GO:0046884: follicle-stimulating hormone secretion (CGA).

The SNPs were used to identify QTLs previously localized in other studies performed in other sheep populations [36,37,38,39], and all QTLs are shown in Table S2. The analysis of the metabolic pathways in which the significant genes participate, showed that 12 genes are associated with some metabolic pathway (Table 1). Analysis of the *DLG1* gene revealed its participation in the Hippo signaling pathway. The *CGA* gene participates in six important metabolic pathways, including the cAMP signaling pathway, GnRH signaling pathway, ovarian steroidogenesis, prolactin signaling pathway, thyroid hormone synthesis, and regulation of lipolysis in adipocytes, which are associated with reproductive processes.

### 3.4. Identification of the Candidate Genes

Functional analysis based on the results of GWAS revealed that the top 10 candidate genes that are involved in the ovary development process in ewes with two lambs at birth were *CLSTN2*, *MTMR2*, *CCDC174*, *NOM1*, *ANKRD11*, *DLG1*, *ALPK3*, *ROBO2*, *CGA*, and *KDM4A*. The relevant genes identified in the present analysis that were reported in other studies to be potentially associated with reproduction processes were *ANKRD11*, *ARID5B*, *DLG1*, *ROBO2*, and *CGA*, as shown in Table 2.

## 4. Discussion

In the present study, we performed a GWAS using the medium-density Illumina OvineSNP50 Genotyping BeadChip. A total of 50,602 SNPs that passed quality control belonging to 47 Pelibuey ewes were used to identify regions associated with litter size. GWAS was able to identify three SNPs significant at 5% at the chromosome-wide level and 54 suggestive SNPs associated with the prolificacy trait. The power of GWAS to detect the true association is determined by many factors such as phenotypic variation, the number of individuals, and allele frequency [52]. The indirect associations are the result of disequilibrium between multiple factors affecting a trait, whereas lack of statistical power can produce spurious associations that are only distantly linked to causal polymorphisms [53]. A low heritability for reproductive traits is associated with a low genetic variability for litter size [54]. A greater heritability of broad sense results in greater −log10 *p*-values, while the number of loci that affect the trait increases along with environmental interactions with an expected decrease in heritability [55]. We used a low number of samples, which could have had an effect on the number of significant SNPs; however, the inflation factor was 1.06 which indicates a low possibility of false positives. When the inflation factor is small (<1.1) it indicates that stratification cannot be excluded as a possibility in real scenarios, to reduce the possibility of confusion due to the population mix [56].

### 4.1. Markers Associated with Prolificacy Traits

Four SNPs identified here may be associated with prolificacy; these are OAR1_204970872.1 and s09883.1 for aseasonal reproduction (ASREP), as well as SNPs OAR2_65914681.1 and s07255.1 for milk yield (MY), and body weight (BW), respectively, as shown in Table 1. In addition, we also report other productive traits identified in other breeds such as muscle weight in carcass (MUSWT), carcass fat percentage (FATP), lean meat yield percentage (LMYP), hot carcass weight (HCWT), internal fat amount (INTFAT), fat weight in carcass (FATWT), and milk lactose yield (MLACT).

An important trait is ASREP, with markers located in two regions (OAR1_204970872.1 (Chr 1: 189,855,910 bp), and s09883.1 (Chr 1: 246,913,454 bp) for seasonal reproduction). In Dorset × East Frisian sheep, seven chromosomes (1, 3, 12, 17, 19, 20, and 24) were identified to harbor putative QTLs for traits associated with ASREP; in chromosome 1, a QTL is associated with the associated with the maximum progesterone level during the pre-breeding season [37]. The increased follicular progesterone secretion comes from the ovary containing the active follicles [57]. In Pelibuey sheep, in the postpartum period, progesterone concentrations rise to luteal phase levels but not to the magnitude of luteinizing hormone (LH) [58]. The markers associated with BW trait are s05724.1 (Chr 1: 56,360,553 bp), OAR1_149400642.1 (Chr 1: 138,085,163 bp), OAR1_150722624.1 (Chr 1: 139,388,579 bp), OAR2_65914681.1 (Chr 2: 61,498,071 bp), OAR3_24559969.1 (Chr 3: 22,729,462 bp), s66102.1 (Chr 3: 33,073,804 bp), OAR3_36221107.1 (Chr3: 33,738,283 bp), s72352.1 (Chr 3: 36,572,814 bp), OAR6_14287930.1 (Chr 6: 11,709,842 bp), s50134.1 (Chr 14: 46,046,471 bp), OAR20_31210307.1 (Chr 20: 28,418,356 bp), and s07255.1 (Chr 23: 47,438,785 bp). In Merino sheep, it was reported that BW is associated with the traits of MUSWT, LMYP, bone weight in carcass (BONE_WT), carcass bone percentage (BONEP), and FATP [36]. In Black Bengal goats, there is a positive relationship between withers height (WH), distance between tuber coxae bones (DTC), BW, and litter size, while physical strength was implicated in an increased likelihood of multiple births in goats bearing multiple fetuses from those bearing a single fetus [59]. 

The SNPs associated with the MY trait are located in OAR2_65914681.1 (Chr 2: 61,498,071 bp), OAR18_22964031.1 (Chr 18: 22,346,018 bp), OAR20_5129052.1 (Chr 20: 5,085,823 bp), OAR20_11623776.1 (Chr 20: 11,249,238 bp), and OAR20_31210307.1 (Chr 20: 28,418,356 bp). In Churra sheep and Lacaune breeds, ewes presenting twin parturitions produced more milk than ewes with single parturitions, and the presence of female lambs had a positive effect on milk yield [60]. In sheep, litter size has a positive correlation with the incidence of breastfeeding and milk production [61]. In Damascus goats, milk production after weaning was hardly affected by post-kidding body weight, and body weight at mating time was linearly related with litter size and weight at subsequent kidding [62], which indicates that it may be associated with litter size in this breed, representing the potential to exploit these genetic traits.

Finally, our study revealed four SNPs s37914.1 (Chr4: 117,719,020 bp), s02969.1 (Chr5: 184,537 bp), OAR15_13905772.1 (Chr15: 13,872,637 bp), and s15631.1 (Chr15: 57,489,437 bp) not previously reported as QTLs on the Jbrowse online page. These four SNPs may have a relationship with the litter size in this breed; however, it is important to carry out analyses of the regions in the corresponding genes (*NOM1, LOC101116985*, *MTMR2*, and *CCDC174*, respectively).

### 4.2. Gene Ontology and Metabolic Pathways

In different studies, identifications of molecular functions and metabolic pathways for genetic loci associated with diseases or traits obtained from GWAS results were carried out [25,63]. We investigated the molecular functions and metabolic processes of the 57 genes identified as a result of the GWAS analysis. Of the 57 genes analyzed, only 13 genes with metabolic pathways and biological functions of reproduction were identified (Table S3). For example, the *DLG1* gene was enriched in six signal pathways; of these, only the Hippo signaling pathway plays a key role in mechanotransduction, providing an understanding of the molecular mechanisms via which cells sense and respond to mechanical signals to regulate cell proliferation and apoptosis for maintaining optimal organ sizes [64]. The Hippo signaling pathway regulates the activation of primordial follicles and increases birth rate, accompanied by increasing levels of 17β-estradiol (E2) and follicle-stimulating hormone (FSH) in mouse [65]. In bovine ovaries, the Hippo pathway transcription co-activators play an important role in GC proliferation and estrogen production, thereby determining normal follicle development [66]. In addition to its role in regulating tissue growth, the pathway was implicated in the control of other biological processes, such as cell-fate determination, mitosis, and pluripotency [67]. The *TRPM6* gene was enriched in the mineral absorption pathway; *TRPM6* is a member of the melastatin-related transient receptor potential (TRPM) subfamily of ion channels, and it is a polypeptide containing both an ion channel pore and a serine/threonine kinase [68]. Studies in mice showed these channels to have a role in whole-body magnesium homeostasis, as well as additional critical functions during early embryogenesis [69]. The *ABCG5* gene was enriched in four pathways, where only the cholesterol metabolism pathway is important, which is utilized for steroid synthesis by ovarian tissue potentially derived from de novo synthesis or cellular uptake of lipoprotein cholesterol [70]. Six important pathways were enriched in the *CGA* gene, which can be the key to understanding the molecular processes that are important for prolificacy. Three of these pathways were the GnRH signaling pathway, ovarian steroidogenesis, and prolactin signaling pathway. The GnRH signaling pathways are activated by signal transduction cascades, which consequently cause the release of gonadotropins, LH, and FSH for gonadal maturation, the onset of puberty, and ovulation [71]. In mice, using GnRH-Ag caused stimulatory effects on ovarian steroidogenesis and follicular development [72]. Ovarian steroidogenesis in sheep is regulated by multiple signals of transduction such as *SHH, WNT*, and *RHO GTPase*, for early folliculogenesis [13]. Prolactin is a hormone that is essential for normal reproduction, and it signals through two types of receptors. The prolactin signaling pathway is initiated by the binding of prolactin with the prolactin receptor (*PRLR*), which is expressed in a variety of tissues [73]. The *HTR1E* gene was enriched in the cAMP signaling pathway, whereby the persistent cAMP signals from internalized LH receptors contribute to transmitting LH effects inside follicle cells and the oocyte [74]. However, these genes can be regulated by extrinsic factors such as nutrition. 

### 4.3. Candidate Gene Identification

After the annotation of the genes, only 10 regions were selected according to their biological functions: s09883.1 (*CLSTN2*), OAR15_13905772.1 (*MTMR2*), s15631.1 (*CCDC174*), s37914.1 (*NOM1*), s57545.1 (*ANKRD11*), OAR1_204970872.1 (*DLG1*), OAR18_22964031.1 (*ALPK3*), OAR1_155672687.1 (*ROBO2*), s62827.1 (*CGA*), and OAR1_18691972.1 (*KDM4A*).

*CLSTN2* (Calsyntenin 2), also known as alcadein-γ, is a neuronal cell surface synaptic protein with an evolutionarily conserved role in learning and memory [75]. In the immature rat uterus, the *CSTN2* gene increased expression in the follicular phase when the level of 17β-estradiol estradiol (E2) increased during the estrous cycle [76]. More recently, in cow, the *CLSTN2* gene was found to play a role related to lipid metabolism, affecting carcass traits [77]. Pensante-Pacheco et al. [78] reported that sheep after calving lose weight and body condition, and lambs born alone were heavier than those born in multiparous litters. Therefore, this gene can act in the metabolic pathways, mainly for the accumulation of energy reserves, allowing animals to survive food shortages that can be used by sheep in reproduction, pregnancy, and lactation; these include changes in synaptic inputs onto GnRH neurons, and the neuroendocrine system signal regulates seasonal reproduction [79]. *MTMR2* (myotubularin-related protein 2), belongs to the family of phosphoinositide phosphatases including several members mutated in neuromuscular diseases or those associated with metabolic syndrome, obesity, and cancer [80]. It was also reported in Schwann cells (SC) [81], lipid metabolic processes [82], and the reproductive process [83]. There is a relationship between E2 and SC, since it promotes SC myelination of regenerated sciatic nerves and can promote SC differentiation through the estrogen receptor beta-extracellular signal-regulated kinase 1 and 2 (ERβ-ERK1/2) signaling pathway in rats [84]. It was reported that the *MTMR2* and *MTMR5* genes are highly expressed in the testicles, especially in germ cells and in Sertoli, and the deactivation of any of these genes produces spermatogenic defects [83]. *CCDC174* (coiled-coil domain-containing 174) is essential for neuronal differentiation; in human, mutations affect psychomotor developmental delay and abducens nerve palsy [85]. The *CCDC174* gene is not yet reported in reproductive processes; however, other members of this family are associated with these processes. For example, 10 coiled-coil domain-containing (CCDC) messenger RNAs (mRNAs) were found in the glandular epithelium (GE), related to pregnancy recognition and establishment [86]. In humans, during normal healthy pregnancy, CCDC proteins significantly increase in exosomes present in maternal plasma with gestational age during the first trimester of pregnancy [87].

*NOM1* (nucleolar protein with MIF4G domain 1) is a member of the MIF4G/MA3 family identified at the chromosome 7q36 breakpoint involved in processes that impact translation [88]. Solomon-Zemler et al. [89] reported that *NOM1* has biological pathways associated with nuclear *IGF1R* (insulin-like growth factor-1 receptor), and inhibition of nuclear *IGF1R* translocation by dansylcadaverine reduced *NOM1* levels in nuclei of MCF7 cells. According to Gunawardena et al. [90], *NOM1* is a *PP1* (protein phosphatase I) nucleolar targeting subunit, which is an essential eukaryotic serine/threonine phosphatase required for many cellular processes, including cell division, signaling, and metabolism. *ANKRD11* (ankyrin repeat domain 11) is a gene directly involved in embryonic and fetal development, which is also associated with maternal nutrition during pregnancy in sheep [91]. *DLG1* (discs large MAGUK scaffold protein 1) is a scaffolding protein that, through interaction with diverse cell partners, participates in the control of key cellular processes such as polarity, proliferation, and migration [92]. Cavatorta et al. [93] reported that the *DLG1* gene encodes a member of the MAGUK protein family involved in the polarization of epithelial cells. In mouse, *DLG1* is expressed at high levels in oocytes and granulosa cells [94]. *ALPK3* (alpha kinase 3) is implicated in a large variety of cellular processes such as protein translation, Mg^2+^ homeostasis, intracellular transport, cell migration, adhesion, and proliferation [95]. A mutation in the *ALPK3* gene in human is associated with cardiomyopathy [96]. The overexpression of *ALPK3* enhances differentiation of murine embryonic carcinoma cells into cardiomyocytes [95]. *ROBO2* (roundabout guidance receptor 2) is important for axon guidance across the midline during central nervous system (CNS) development [97]. In sheep, the *ROBO2* gene is essential during the early stages of follicle formation, as well as during primordial follicle maturation-determining processes in ovary development [98], and expression may be regulated by additional factors in the ovary and steroid hormones in other reproductive tissues [99]. *CGA* (glycoprotein hormone, alpha polypeptide) is the α subunit of glycoprotein hormones, with an important role in the development and function of thyroid and gonads [100]. Two subunits were reported in the FSH; *FSHα* and *FSHβ* play key roles in female reproduction, including boars [101,102], where each subunit regulates a variety of functions of the hormone including folding, heterodimerization, secretion, circulatory survival, and bioactivity [103]. Previous reports indicated that *FSHα* and *FSHβ* mRNAs were detected only in the pituitary tissue of boar [101]. *KDM4A* (lysine demethylase 4A) is a lysine demethylase with specificity toward di- and tri-methylated lysine 9 and lysine 36 of histone H3 (H3K9me2/me3 and H3K36me2/me3) [104]; a previous study reported that *KDM4A* is a maternal factor that plays a key role in embryo survival, the implantation process in mice, and fertility [104]. All these genes may have an important role in cellular processes such as follicular development associated with the regulation of the litter size identified in the Pelibuey sheep. In addition to the 10 SNPs significant at the chromosome-wide level associated with prolificacy, we also report three new uncharacterized proteins were identified in this study. These were OAR1_149400642.1 (Chr 1: 138,085,163 bp) (*p* < 0.0000837), s11062.1 (Chr 14: 13,674,017 bp) (*p* < 0.00000729), and OAR20_31210307.1 (Chr 20: 28,418,356 bp) (*p* < 0.00000211), located in genes *LOC101114740*, *LOC105616840*, and *LOC101117202*, respectively (Table S1). However, the information for these genes is non-existent; thus, it is necessary to expand the research to obtain thorough knowledge associated with the reproduction of sheep.

## 5. Conclusions

The GWAS performed was able to identify 57 SNPs associated with litter size in the Pelibuey breeds. The candidate genes associated with litter size in Pelibuey that may be involved in the reproduction process are *CLSTN2, MTMR2, CCDC174, NOM1, ANKRD11, DLG1, ALPK3, ROBO2, CGA,* and *KDM4A*. In addition, we also report four SNPs not previously documented whose QTLs were previously reported in sheep: s37914.1 (Chr 4: 117,719,020 bp), s02969.1 (Chr 5: 184,537 bp), OAR15_13905772.1 (Chr 15: 13,872,637 bp), and s15631.1 (Chr 19: 57,489,437 bp). The presence of SNPs commonly present in prolific wool sheep breeds such as for the transforming factor group β (TGF β) was not reported in this hair sheep, which may confirm that prolificacy in Pelibuey sheep can be controlled via different underlying genetic mechanisms, such as candidate genes related to reproductive seasonality, milk yield, and body weight. Further validation studies can elucidate the gene network, enabling its incorporation in sheep breeding programs in order to obtain ewes with higher reproductive efficiency.

## Figures and Tables

**Table 1:** SNPs identified by chromosome genome-wide association, traits, and biological pathway association with prolificacy.

SNP ID	Chr	Position (bp)	Gene Name	Gene Description	Traits	Signal Pathway
s71757.1	1	51,963,826	*ST6GALNAC3*	ST6 *N*-acetylgalactosaminide alpha-2 6-sialyltransferase 3	MUSWT, LMYP, BONE_WT, BONEP, FATP [36]	Glycosphingolipid biosynthesis
OAR1_155672687.1	1	189,855,910	*ROBO2*	Roundabout guidance receptor 2	MUSWT, LMYP, BONE_WT, BONEP, FATP [36]	Axon guidance
OAR1_204970872.1	1	189,855,910	*DLG1*	Discs large MAGUK scaffold protein 1	ASREP [37], SAOS [43], LMYP, FATP, BONE_WT, MUSWT [36]	Hippo signaling pathway; tight junction; T-cell receptor signaling pathway
s09883.1	1	246,913,454	*CLSTN2*	Calsyntenin 2	ASREP [37], TFEC_1 [44], FATP [36], FCURV [45]	
OAR2_65914681.1	2	61,498,071	*TRPM6*	Transient receptor potential cation channel subfamily M member 6	HCWT [46], MF [39], MFY, PP, MY [38], BW [36]	Mineral absorption
OAR2_95966123.1	2	89,499,669	*COL11A1*	Collagen type XI alpha 1 chain	SCS [46], PP [38], LATRICH2 [47], HCWT [36], MF [38]	Protein digestion and absorption
OAR3_85112203.1	3	80,398,784	*ABCG5*	ATP-binding cassette subfamily G member 5	INTFAT [36], MCLA [48], SL [49]	ABC transporters; fat digestion and absorption; bile secretion; cholesterol metabolism
OAR3_104545117_X.1	3	98,126,615	*HTRA2*	HtrA serine peptidase 2	FECZ [50], MCLA [48], SL [49], INTFAT [36]	Apoptosis
s62827.1	8	49,878,423	*CGA*	Glycoprotein hormones alpha polypeptide	LATRICH2 [47], INTFAT [36], FECGEN [51]	cAMP signaling pathway; GnRH signaling pathway; ovarian steroidogenesis; prolactin signaling pathway; thyroid hormone synthesis; regulation of lipolysis in adipocytes
OAR8_53593379.1	8	49,981,252	*HTR1E*	5-hydroxytryptamine receptor 1E	LATRICH2 [47], INTFAT [36], FECGEN [51]	cAMP signaling pathway; neuroactive ligand–receptor interaction; serotonergic synapse; taste transduction
OAR9_36598045.1	9	34,604,487	*ATP6V1H*	ATPase H^+^ transporting V1 subunit H	HCWT, LMA [36]	Oxidative phosphorylation; metabolic pathways; phagosome; mTOR signaling pathway; synaptic vesicle cycle
OAR15_13905772.1	15	13,872,637	*MTMR2*	Myotubularin related protein 2		Inositol phosphate metabolism; metabolic pathways; phosphatidylinositol signaling system
s07255.1	23	47,438,785	*ST8SIA5*	ST8 alpha-*N*-acetyl-neuraminide alpha-2 8-sialyltransferase 5	IGA [51], FATP, LMYP, HCWT, BW, FATWT [36], MFY, MY [38]	Glycosphingolipid biosynthesis; metabolic pathways
s08197.1	25	40,382,673	*GRID1*	Glutamate ionotropic receptor delta type subunit 1	SL, MFDIAM, CVFD_PRI [49]	Neuroactive ligand–receptor interaction

Chr, chromosome; ID, identifier; bp, base pairs; MUSWT, muscle weight in carcass; LMYP, lean meat yield percentage; BONE_WT, bone weight in carcass; BONEP, carcass bone percentage; FATP, carcass fat percentage; ASREP, aseasonal reproduction; SAOS, *Salmonella abortus ovis* susceptibility; TFEC_1, *Trichostrongylus colubriformis* FEC; FCURV, fiber curvature; HCWT, hot carcass weight; MF, milk fat percentage; MFY, milk fat yield; PP, milk protein percentage; MY, milk yield; BW, body weight; SCS, somatic cell score; LATRICH, *Trichostrongylus* adult and larva count; INTFAT, internal fat amount; MCLA, meat-conjugated linoleic acid content; SL, staple length; FECGEN, fecal egg count; LMA, longissimus muscle area; IGA, immunoglobulin A level; FATWT, fat weight in carcass; MFDIAM, mean fiber diameter; CVFD_PRI, primary fiber diameter coefficient of variance.

**Table 2:** The SNPs identified by GWAS for prolificacy in Pelibuey sheep.

Chr	SNP	Position (bp)	A1	A2	F_A	F_U	MAF	*p*-Unadjusted	Chis-q	Gene Annotated
1	s09883.1	246,913,454	A	G	0.1458	0.587	0.3617	8.61 × 10^−6^	19.8	*CLSTN2*
15	OAR15_13905772.1	13,872,637	A	G	0.04167	0.3261	0.1809	0.0003417	12.83	*MTMR2*
19	s15631.1	57,489,437	A	C	0.5208	0.1739	0.3511	0.0004272	12.41	*CCDC174*
4	s37914.1	117,719,020	G	A	0.6667	0.3043	0.4894	0.0004434	12.34	*NOM1*
14	s57545.1	13,903,063	T	C	0.5417	0.1957	0.3723	0.0005225	12.03	*ANKRD11*
1	OAR1_204970872.1	189,855,910	T	C	0.3542	0.06522	0.2128	0.0006221	11.71	*DLG1*
18	OAR18_22964031.1	22,346,018	C	T	0.1875	0.5217	0.3511	0.0006891	11.52	*ALPK3*
1	OAR1_155672687.1	144,029,243	C	T	0.625	0.2826	0.4574	0.0008655	11.1	*ROBO2*
8	s62827.1	49,878,423	A	G	0.08333	0.3696	0.2234	0.0008669	11.09	*CGA*
1	OAR1_18691972.1	18,481,816	T	C	0.25	0.587	0.4149	0.0009179	10.99	*KDM4A*

Chr, chromosome; bp, base pairs; A1, minor allele 1; A2, major allele 2; F_A, allele 1 frequency among cases; F_U, allele 1 frequency among controls; MAF, minor allele frequency.

## Supplementary Materials

The following are available online at https://www.mdpi.com/2076-2615/10/3/434/s1: Table S1: 57 SNPs considered as candidate markers and genes mapped; Table S2: Regions of chromosome in SNPs identified in the present study and QTLs previously reported for traits different in other sheep breeds; Table S3: KEGG pathway analysis in genes with chromosome-wide level association.
